# Both Allene Oxide Synthases Genes Are Involved in the Biosynthesis of Herbivore-Induced Jasmonic Acid and Herbivore Resistance in Rice

**DOI:** 10.3390/plants10030442

**Published:** 2021-02-26

**Authors:** Jiamei Zeng, Tongfang Zhang, Jiayi Huangfu, Ran Li, Yonggen Lou

**Affiliations:** 1 State Key Laboratory of Rice Biology & Ministry of Agriculture Key Lab of Molecular Biology of Crop Pathogens and Insects, Institute of Insect Sciences, Zhejiang University, Hangzhou 310058, China; tsengchiamei@zju.edu.cn (J.Z.); zhangtf@swu.edu.cn (T.Z.); luckyfjy@163.com (J.H.); rli05@zju.edu.cn (R.L.); 2 College of Food Science, Southwest University, Chongqing 400715, China

**Keywords:** allene oxide synthase, jasmonic acid, rice, herbivore resistance, salicylic acid, hydrogen peroxide

## Abstract

Allene oxide synthase (AOS) is the second enzyme in the biosynthesis of the plant defensive hormone jasmonic acid (JA). In rice, there are two AOSs, OsAOS1 and OsAOS2. However, the role of these two AOS genes in herbivore-induced defenses in rice remains unidentified. We cloned the two rice AOS genes and observed that the transcript level of both *OsAOS1* and *OsAOS2* was enhanced by mechanical wounding, the infestation of the striped stem borer (SSB) (*Chilo suppressalis*) or brown planthopper (BPH) (*Niaparvata lugens*), and treatment with JA; however, *OsAOS1* responded more rapidly to SSB infestation and JA treatment than did *OsAOS2*. The antisense expression of *OsAOS1* (as-*aos1*) or *OsAOS2* (as-*aos2*) decreased levels of SSB- or BPH-induced JA, which, in turn, reduced the production of SSB-induced trypsin protease inhibitor (TrypPI) and volatiles as well as the resistance of rice to SSB. In contrast, BPH preferred to feed and oviposit on wild-type (WT) plants over as-*aos1* and as-*aos2* plants. Moreover, the survival of BPH nymphs on as-*aos1* or as-*aos2* lines was significantly lower than on WT plants. The increased resistance of as-*aos1* or as-*aos2* plants to BPH correlated with higher levels of BPH-induced H_2_O_2_ and SA. These results indicate that *OsAOS1* and *OsAOS2* are both involved in herbivore-induced JA biosynthesis and play a vital role in determining the resistance of rice to chewing and phloem-feeding herbivores.

## 1. Introduction

When infested by herbivorous insects, plants recognize herbivore-associated molecular patterns and then initiate defense-related signaling pathways; these activated pathways, thus, induce the expression of defensive genes and the biosynthesis of defensive compounds, which, in turn, enhance the resistance of plants to herbivorous insects [1,2]. In these signaling pathways, the jasmonates-mediated pathway plays a central role [3,4,5]. The biosynthesis of jasmonates starts with the transformation from α-linolenic acid (α-LeA) (18:3) released from chloroplast membranes and continues to 13*S*-hydroperoxy-(9*Z*,11*E*,15*Z*)-octadecatrienoic acid (13-HPOT) catalyzed by 13-lipoxygenase (LOX). Subsequently, the 13-HPOT is oxidized by an allene oxide synthase (AOS) to form an unstable epoxide, which is cyclized to the 12-oxo phytodienoic acid (12-OPDA) by an allene oxide cyclase (AOC). Finally, OPDA is reduced/converted to jasmonic acid (JA) by OPDA reductase3 (OPR3) and three cycles of β-oxidation in the peroxisomes [6,7]. JA may then form distinct jasmonates via different metabolic conversions. Therefore, it is clear that the enzyme AOS is a regulatory point in the biosynthesis of jasmonates [8,9], which play vital roles in plant development and responses to abiotic and biotic stresses [10].

Thus far, many AOS genes have been cloned and characterized in various dicots and monocots [11,12]. The number of genes encoding AOS varies with plant species. For example, a single AOS gene has been described in *Arabidopsis* [13], two AOSs have been found in barley (*Hordeum vulgare*) [14] and tomato (*Solanum lycopersicum*) [15,16], and three AOSs have been identified in potato (*S. tuberosum*) [17]. Despite conflicting reports on the number of AOS genes in rice (one [18], two [19], four [20], or five [21]), a series of detailed database searches followed by functionality tests has convincingly shown that there are only two AOS genes whose encoding proteins localize in the chloroplast [11].

Rice, one of the most important staple food crops in the world, suffers heavily from insect pests [22], among which striped stem borer (SSB) *Chilo suppressalis* (Lepidoptera: Pyralidae), a chewing herbivore, and brown planthopper (BPH) *Nilaparvata lugens* (Stål) (Hemiptera: Delphacidae), a piercing-sucking feeder, are two of the most important [23]. It has been well documented in rice that herbivorous insect infestation activates a variety of defensive signaling pathways mediated mainly by JA, salicylic acid (SA), ethylene, and H_2_O_2_; these signaling pathways facilitate the accumulation of trypsin proteinase inhibitors (TrypPIs) and the release of herbivore-induced plant volatiles (HIPVs), thereby enhancing the direct and indirect resistance of rice to herbivores [24,25]. The expression levels of both *OsAOS1* and *OsAOS2* are significantly induced following the infection of rice blast fungus (*Magnaporthe grisea*) (Magnaporthales: Magnaporthaceae) [18,26]. Overexpression of *OsAOS2* enhances the activation of pathogenesis-related (PR) genes and increases the resistance of rice to *M. grisea* [26]. However, the role of OsAOSs in herbivore-induced defense responses in rice remains largely unknown.

In this study, we cloned the two rice *AOSs* and characterized their roles in herbivore-induced defenses in rice. We found that the expression of *OsAOS1* and *OsAOS2* was induced by mechanical wounding, herbivore infestation, and JA treatment. Both *OsAOS1* and *OsAOS2* positively regulate the production of herbivore-induced JA, volatiles, and TrypPIs but negatively modulate the biosynthesis of herbivore-induced SA and H_2_O_2_. Moreover, silencing *OsAOS1* or *OsAOS2* reduced the resistance of rice to the chewing herbivore SSB but enhanced the resistance to the piercing-sucking herbivore BPH. These findings demonstrate that both OsAOS1 and OsAOS2 play an important role in the biosynthesis of herbivore-induced JA and in the resistance of rice to herbivores.

## 2. Results

### 2.1. Both OsAOS1 and OsAOS2 Were Induced by Mechanical Wounding, Herbivore Infestation, and JA but Have Different Patterns

We cloned the full-length cDNA of the sequenced rice AOS genes, *OsAOS1* (TIGR ID Os03g55800) and *OsAOS2* (Os03g12500), using reverse transcription polymerase chain reaction (RT-PCR) (Figure S1). The first AOS gene has an open reading frame (ORF) of 1,539 bp and encodes 513 amino acids; its predicted molecular mass is 56.50 kDa and its pI is 9.52. The second AOS gene has an ORF of 1,437 bp and encodes 479 amino acids; its predicted molecular mass is 52.27 kDa and its pI is 8.26. Sequence alignments revealed that both OsAOS1 and OsAOS2 shared 65.75% and 53.70% identity, respectively, in nucleotide sequence and amino acid sequence (Figure S2).

**Figure 1:**
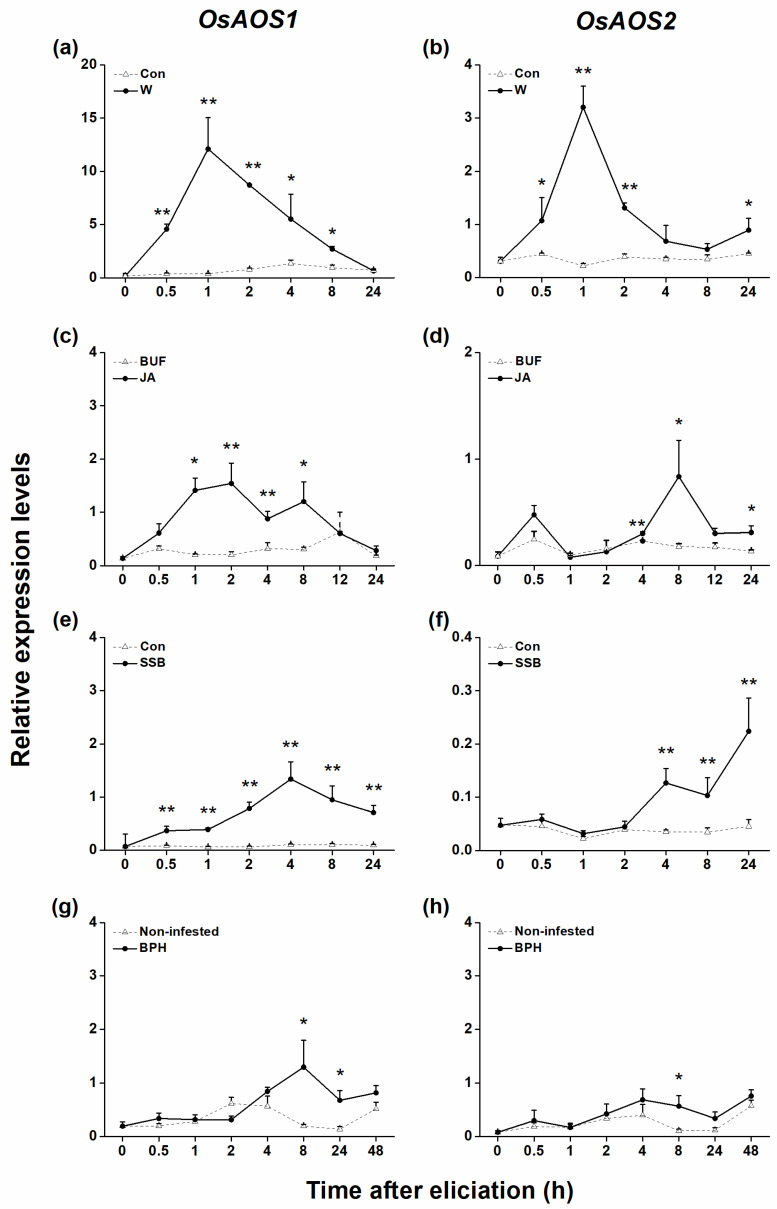
Transcript levels of *OsAOS1* and *OsAOS2* in rice after various treatments. Mean expression levels (relative to expression levels of *OsACT*, +SE, *n* = 5) of *OsAOS1* (**a**,**c**,**e**,**g**) and *OsAOS2* (**b**,**d**,**f**,**h**) in rice leaf sheaths that were treated by mechanically wounded (W, **a**,**b**), jasmonic acid (JA, **c**,**d**), or infested by rice striped stem borer (SSB, **e**,**f**) or brown planthopper (BPH, **g**,**h**). BUF, sodium phosphate buffer; Non-infested, plants with an empty cylinder; Con, control plants. Asterisks indicate significant differences between treatments and controls (* *p* < 0.05, ** *p* < 0.01, Student’s *t*-test).

Quantitative real-time (qRT)-PCR analysis revealed that constitutive transcript levels of both *OsAOS1* and *OsAOS2* in rice leaf sheaths were low. When plants were mechanically wounded, infested with herbivores, or treated with JA, transcript levels of the two AOS genes increased with different patterns (Figure 1a–f): generally, *OsAOS1* responded to these treatments more strongly than did *OsAOS2*; moreover, SSB infestation and JA treatment induced the expression of *OsAOS2* slowly (≥4 h after treatment) but induced *OsAOS1* quickly. BPH infestation also elicited the accumulation of *OsAOS1* and *OsAOS2* transcripts but slowly and weakly (Figure 1g,h). SA treatment did not induce the expression of either *OsAOS1* or *OsAOS2* (Figure S3). These data suggest that although *OsAOS1* and *OsAOS2* exhibited different induced expression profiles, both seem to be involved in the herbivore-induced JA signaling pathway in rice.

### 2.2. Silencing OsAOS1 and OsAOS2

We constructed a pCAMBIA-1301 transformation vector carrying reverse fragments of *OsAOS* (Figure S4) and created transgenic rice plants using *Agrobacterium tumefaciens*-mediated transformation. By β-glucuronidase (GUS) staining and hygromycin resistance selection, we obtained six T_2_ homozygous lines, including three *OsAOS1*-silenced lines (as-*aos1* lines: as1-3, as1-5, and as1-10) and three *OsAOS2*-silenced lines (as-*aos2* lines: as2-10, as2-20, and as2-58) (Figure S5). Transcriptional analysis showed that SSB-induced transcript levels of *OsAOS1* and *OsAOS2* in as-*aos1* lines (as1-3, as1-5, and as1-10) and as-*aos2* lines (as2-10, as2-20, and as2-58) were only 38.02%, 16.61%, and 38.34%, and 36.18%, 24.91%, and 27.11% of those in wild-type (WT) plants 1 h after SSB infestation, respectively (Figure S6). In rice, the gene whose nucleotide sequence has the highest similarity to *OsAOS1* is *OsAOS2* (65.75%, Figure S2) and vice versa. Transcription analysis revealed that both *OsAOS1* and *OsAOS2* antisense constructs silenced the transcript accumulation of the targeted gene but not the other (Figure S7), suggesting that the specificity of the RNAi sequence for *OsAOS1* or *OsAOS2* is high. No obvious difference in growth phenotype was observed between WT plants and transgenic lines during their entire development (Figure S8).

### 2.3. Both OsAOS1 and OsAOS2 Mediate Herbivore-Induced JA and SA Biosynthesis

**Figure 2:**
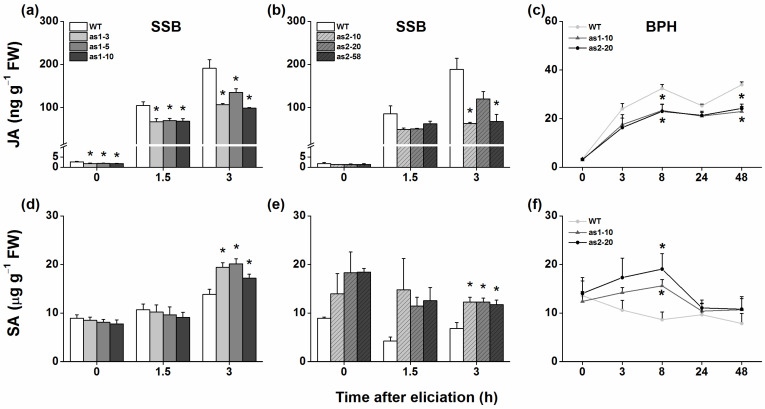
Levels of JA and SA in as-*aos* lines and WT plants that were infested by SSB or BPH. Mean levels (+SE, *n* = 5) of JA (**a**–**c**) and SA (**d**–**f**) levels in leaf sheaths of as-*aos*1 (as1-3, as1-5, and as1-10), as-*aos2* (as2-10, as2-20, and as2-58) and WT plants that were individually infested by a third-instar SSB larva or 15 gravid BPH females. FW, fresh weight. Asterisks indicate significant differences between as-*aos* lines and WT plants at the indicated times (* *p* < 0.05, Tukey’s honest significant difference (HSD) post-hoc test).

JA and SA play an important role in herbivore-induced defense responses in rice [25]. Hence, we asked if silencing *OsAOS1* or *OsAOS2* influences the production of the basal and herbivore-induced JA and SA in rice. Phytohormone analysis revealed that basal and SSB-induced levels of JA in both as-*aos1* and as-*aos2* lines were lower than those in WT plants, although the difference in basal and induced (1.5 h after SSB infestation) levels of JA between WT plants and as-*aos2* lines was not significant (Figure 2a,b). Similarly, silencing *OsAOS1* or *OsAOS2* also decreased BPH-induced levels of JA in plants 8 h and 48 h after BPH infestation (Figure 2c). In contrast, no difference was found in constitutive SA levels between WT plants and transgenic lines; however, 3 h after SSB infestation or 8 h after BPH infestation, SA levels in as-*aos1* and as-*aos2* lines were significantly higher than those in WT plants (Figure 2d–f).

### 2.4. OsAOS1 and OsAOS2 Positively Regulates TrypPI Activity, Volatile Emmission, and Rice Resistance to SSB

**Figure 3:**
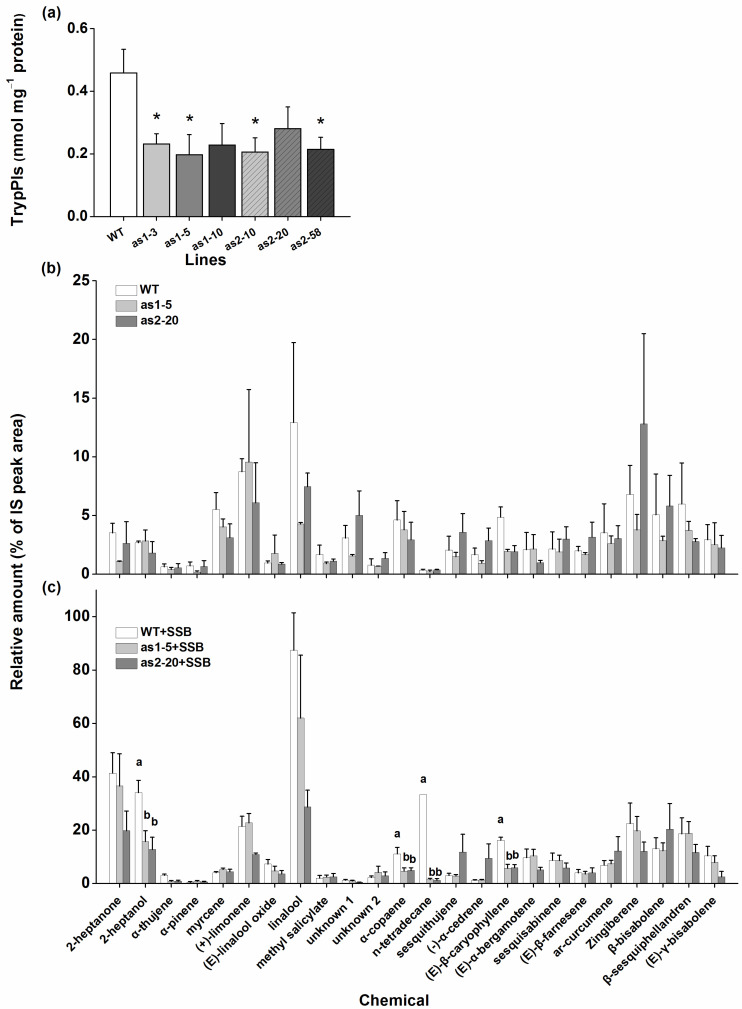
OsAOS1 and OsAOS2 regulate SSB-induced TrypPI activity and volatile emission. (**a**) Mean TrypPI activity (+SE, *n* = 5) in as-*aos1*, as-*aos2*, and WT plants that were individually infested by a third-instar SSB larva for 3 days. Mean amounts (% of IS peak area, +SE, *n* = 5) of volatiles emitted from as-*aos1* (as1-5), as-*aos2* (as2-20), and WT plants that were not manipulated (**b**) or were individually infested with a third-instar SSB larva for 24 h (**c**). Asterisks and letters indicate significant differences in as-*aos* lines compared with WT plants (* *p* < 0.05, Tukey’s HSD post-hoc test).

TrypPIs are important direct defensive compounds against SSB in rice [25]. To investigate the role of OsAOS1 and OsAOS2 in regulating the activity of TrypPIs, we measured TrypPI activity in WT plants and transgenic lines 3 days after SSB infestation. Compared with WT plants, both as-*aos1* and as-*aos2* lines showed less SSB-induced activity in TrypPIs (Figure 3a).

It has been reported that SSB caterpillar infestation induces the production of rice volatiles that attract the natural enemies of SSB caterpillars [27]. Thus, we collected and analyzed the volatiles released from WT and transgenic plants that were infested by SSB or not. The results showed that constitutive levels of volatiles emitted from WT, as-*aos1* and, as-*aos2* plants were similar (Figure 3b). However, when plants were infested by SSB, the total amount of volatiles released from as-*aos1* and as-*aos2* plants was lower than that emitted from WT plants, although the production of volatiles from all these plants were induced by SSB infestation (Figure 3b,c; Table S1). Moreover, levels of four compounds, 2-heptanol, α-copaene, n-tetradecane, and (*E*)-β-caryophyllene were significantly lower in as-*aos1* and as-*aos2* plants than in WT plants (Figure 3c).

**Figure 4:**
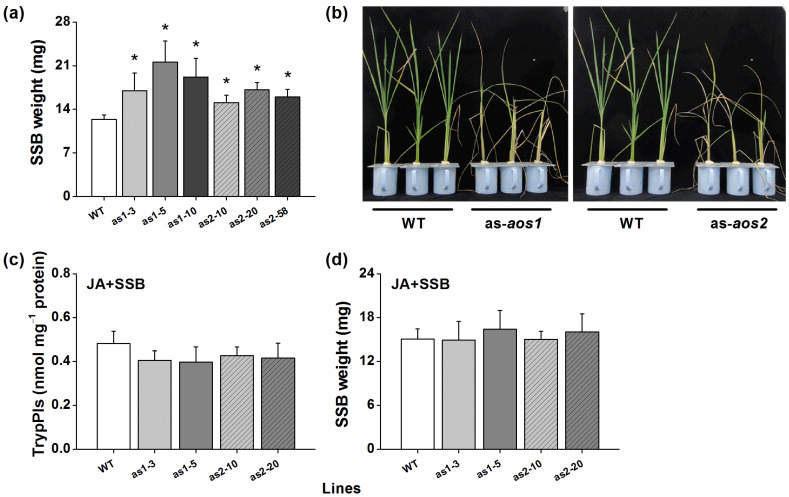
OsAOS1 and OsAOS2 positively regulate the direct resistance of rice to SSB. (**a**) Mean larval weight (+SE, *n* = 30) of SSB fed on as-*aos1* (as1-3, as1-5, and as1-10), as-*aos2* (as2-10, as2-20, and as2-58), and WT plants for 12 days. (**b**) Damaged phenotypes of as-*aos1*, as-*aos2*, and WT plants that were individually infested by a third-instar SSB larva for 10 days. (**c**) Mean TrypPI activity (+SE, *n* = 5) in as-*aos1*, as-*aos2*, and WT plants that were first individually sprayed with 2 mL of JA (100 μg mL^−1^) in the sodium phosphate buffer for 1 day, followed by a third-instar SSB larvae infestation for 3 days. (**d**) Mean larval mass (+SE, *n* = 30) of SSB 12 days after they fed on as-*aos1*, as-*aos2*, and WT plants that were individually treated with JA as stated above. Asterisks indicate significant differences in as-*aos* lines compared with WT plants (* *p* < 0.05, Tukey’s HSD post-hoc test).

SSB caterpillars gained more mass on as-*aos1* and as-*aos2* lines than on WT plants (Figure 4a). Consistent with this finding, as-*aos1* and as-*aos2* plants were damaged more severely by SSB than were WT plants (Figure 4b). To determine whether the reduction in JA level in as-*aos1* and as-*aos2* lines was sufficient to explain the reduction in TrypPI activity and SSB resistance in rice, we measured TrypPI activity in, and SSB caterpillar mass on, as-*aos1* and as-*aos2* lines complemented with JA. We observed that when plants were treated with JA, SSB-induced TrypPI levels in as-*aos1* and as-*aos2* lines were similar to those in WT plants (Figure 4c); moreover, SSB caterpillars fed on JA-treated as-*aos1* and as-*aos2* lines gained the same weight as those fed on JA-treated WT plants (Figure 4d).

### 2.5. OsAOS1 and OsAOS2 Negatively Modulate H_2_O_2_ Accumulation and Rice Resistance to BPH

**Figure 5:**
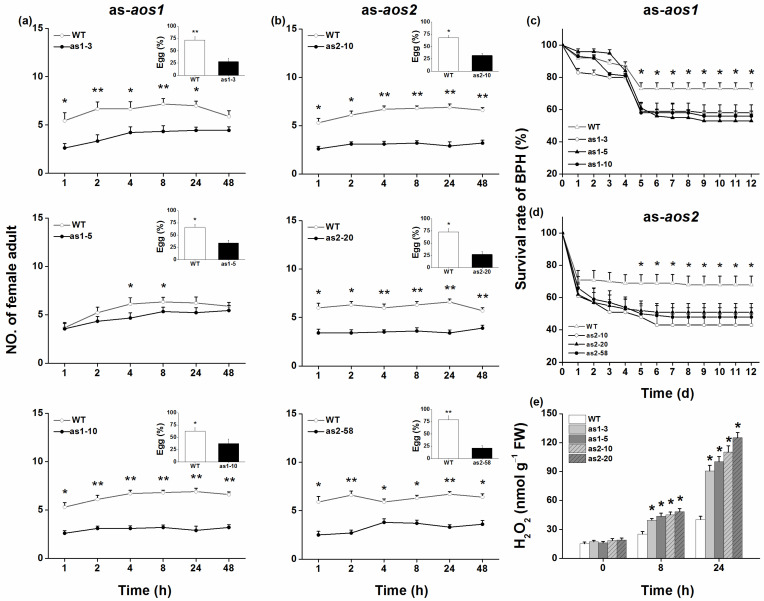
OsAOS1 and OsAOS2 negatively modulate the resistance of rice to BPH. (**a**,**b**) Mean number of gravid BPH females per plant (+SE, *n* = 10) on pairs of plants (WT versus as-*aos1* (as1-3, as1-5, and as1-10) (**a**) or as-*aos2* (as2-10, as2-20, and as2-58) (**b**), respectively), 1–48 h after plant pairs were exposed to 13 insects. Inserts: Mean percentage (+SE, *n* = 10) of BPH eggs per plant on pairs of plants as stated above. (**c**,**d**) Mean survival rates (+SE, *n* = 10) of BPH newly hatched nymphs fed on as-*aos1*, as-*aos2*, and WT plants, 1–12 days after the nymphs were placed on plants. (**e**) Mean concentrations (+SE, *n* = 5) of H_2_O_2_ in as-*aos1*, as-*aos2*, and WT plants that were individually infested by 12 gravid BPH females for 8 and 24 h. Asterisks indicate significant differences in as-*aos* lines compared with WT plants (* *p* < 0.05, ** *p* < 0.01, Student’s *t*-tests (**a**,**b**) or Tukey’s HSD post-hoc test (**c**–**e**)).

We also tested whether silencing *OsAOS1* or *OsAOS2* influences the resistance of rice to BPH. When as-*aos1* or as-*aos2* lines and WT plants were exposed to a BPH colony, gravid BPH females preferred to feed and lay eggs on WT plants (Figure 5a,b, and insets). Moreover, the survival of BPH nymphs fed on as-*aos1* or as-*aos2* lines was lower than the survival of those fed on WT plants (Figure 5c,d).

H_2_O_2_ signaling positively modulates the resistance of rice to BPH [25,28,29]. Hence, we measured H_2_O_2_ levels in WT, as-*aos1*, and as-*aos2* plants when they were infested by gravid BPH females. The results revealed that levels of BPH-induced H_2_O_2_ were significantly higher in as-*aos1* and as-*aos2* lines than in WT plants (Figure 5e).

## 3. Discussion

In this study, we evaluated the role of two *OsAOS* genes in the biosynthesis of herbivore-induced JA and the resistance of rice to herbivores. Several lines of evidence suggest that both OsAOS1 and OsAOS2 play an important role in these processes. First, both *OsAOS1* and *OsAOS2* were induced by SSB caterpillar infestation, gravid BPH female infestation, mechanical wounding, or JA treatment but not SA treatment (Figure 1 and Figure S2). Second, silencing *OsAOS1* or *OsAOS2* reduced levels of herbivore-induced JA and enhanced levels of SA (Figure 2), which in turn decreased the production of SSB-induced TrypPIs and volatiles (Figure 3), and the resistance of rice to SSB (Figure 4a,b). Third, supplementing with JA on as-*aos1* and as-*aos2* plants restored the activity of TrypPIs and the resistance to SSB (Figure 4c,d). Fourth, silencing *OsAOS1* or *OsAOS2* enhanced levels of BPH-induced H_2_O_2_ and the resistance of rice to BPH (Figure 5). These data suggest that both OsAOS1 and OsAOS2 are involved in the biosynthesis of herbivore-induced JA and that the JA signaling pathway plays a key role in regulating the resistance of rice to SSB and BPH directly or indirectly via modulating other signaling pathways.

Consistent with previous reports in many plant species, such as *Arabidopsis*, that the expression of *AOS* is rapidly induced by herbivore infestation or mechanical wounding [13,30], we found that both *OsAOS1* and *OsAOS2* were induced by mechanical wounding, herbivore (SSB or BPH) infestation, and JA treatment (Figure 1). Exogenous application of SA has been reported to affect *AOS* transcript levels differently in different plant species. In barley, for example, SA treatment did not influence the expression of either *AOS1* or *AOS2* [14], whereas in *Arabidopsis*, SA treatment upregulated the transcript level of *AOS* [31]. In this study, we observed that the exogenous application of SA had no effect on transcript levels of either *OsAOS1* or *OsAOS2* within 24 h (Figure S3). This finding was consistent with the result reported in Agrawal et al. [18], who found that SA-induced *OsAOS2* expression was detectable only at 48 h or more after treatment.

Mei et al. [26] reported that the overexpression of *OsAOS2* increases the JA level in rice. Here, we observed that silencing *OsAOS1* or *OsAOS2* decreased levels of SSB- or BPH-induced JA (Figure 2a–c). These findings demonstrate that, consistent with results reported in other plant species [32], both rice AOS genes, OsAOS1 and OsAOS2, are essential in the production of JA. Intriguingly, silencing *OsAOS1* decreased not only constitutive levels of JA but also levels of SSB-induced JA 1.5 and 3 h after SSB infestation, whereas silencing *OsAOS2* reduced levels of SSB-induced JA only 3 h after SSB infestation (Figure 2a,b); moreover, silencing *OsAOS1* or *OsAOS2* had a similar effect on gravid BPH female-induced levels of JA (Figure 2c). This different effect of *OsAOS1* and *OsAOS2* on herbivore-induced JA biosynthesis well-matched their expression patterns: *OsAOS1* responded to SSB infestation and JA treatment more quickly than did *OsAOS2*, whereas both genes were similarly responsive to BPH infestation (Figure 1). These results indicate that *OsAOs1* and *OsAOS2* play an important but slightly different role in the biosynthesis of herbivore-induced JA.

In addition to JA, we also found that silencing *OsAOS1* or *OsAOS2* increased levels of SSB- or BPH-induced SA (Figure 2d–f) and of BPH-induced H_2_O_2_ (Figure 5e). Antagonistic crosstalk between JA and SA has been well documented in many plant species, including rice [33,34]. Moreover, in rice, it has been reported that impaired JA biosynthesis enhances the level of BPH-induced H_2_O_2_ [25,28]. Hence, the increase in levels of SSB- or BPH-induced SA and of BPH-elicited H_2_O_2_ in as-*aos1* and as-*aos2* lines is probably due to the decrease in levels of herbivore-induced JA in these plants.

In rice, the JA signaling pathway has been reported to positively modulate the biosynthesis of many defensive compounds, such as TrypPIs and volatiles, and the resistance of rice to herbivores [25,34]. Moreover, TrypPIs and herbivore-induced plant volatiles are important direct and indirect defensive compounds in the resistance of rice to SSB [25,27,28,34]. Therefore, the decrease in levels of SSB-induced TrypPIs and volatiles in as-*aos1* and as-*aos2* lines, compared to WT plants, occurs mainly because levels of SSB-elicited JA in these lines are low. Moreover, the attenuated SSB resistance in as-*aos1* and as-*aos2* plants, compared to in WT plants, is probably due to their relatively lower TrypPI activity. The fact that supplementation with JA restores the activity of induced TrypPIs and the resistance to SSB in as-*aos1* and as-*aos2* lines also supports these inferred conclusions stated above. Whether changed SSB-induced rice volatiles also directly influence the performance of SSB and thereby influence the resistance of rice remains to be elucidated.

Unlike the result that silencing *OsAOS1* or *OsAOS2* decreased the resistance of rice to SSB caterpillars, silencing *OsAOS1* or *OsAOS2* enhanced the resistance of rice to BPH (Figure 5a–d). These results confirmed our previous results showing that plants with JA pathways impaired by silencing a LOX gene, *OsHI-LOX*, were more resistant to BPH than WT plants [25]. Both SA [35] and H_2_O_2_ [25,29] pathways positively modulate the resistance of rice to BPH. Hence, the increase in resistance to BPH in as-*aos1* and as-*aos2* lines might be related to higher levels of herbivore-induced SA and H_2_O_2_ in these lines compared to WT plants.

In summary, our results demonstrate that the two rice AOS genes, *OsAOS1* and *OsAOS2*, are involved in the biosynthesis of wounding- and herbivore-induced JA, a process that in turn plays an important role in mediating the resistance of rice to chewing and phloem-feeding herbivores directly or indirectly by modulating other signaling pathways.

## 4. Materials and Methods

### 4.1. Plants and Insects

In this study, the rice genotypes used were Xiushui 11 (WT) and transgenic lines as-*aos1* and as-*aos2* (see details below). Pre-germinated seeds of all the lines were cultured in plastic bottles (height 10 cm, diameter 8 cm) in the greenhouse (27 ± 1 °C, 14-L:10-D). One-week-old seedlings were transferred to 20 L hydroponic boxes with a rice nutrient solution [36]. After 30–35 days, seedlings were transplanted to individual plastic pots containing 500 mL hydroponic nutrient solution. Plants were used for experiments 4–5 days after transplantation. Colonies of BPH and SSB were originally collected from rice fields in Hangzhou, China, and maintained on rice seedlings of Xianyou 63, a variety susceptible to BPH and SSB, in a controlled climate room at 27 ± 1 °C, 12-h light phase, and 80% relative humidity.

### 4.2. Cloning and Sequence Analysis of OsAOS1 and OsAOS2

The full-length cDNAs of *OsAOS1* and *OsAOS2* were amplified by PCR. The primers (Table S2) were designed based on the sequence of two rice AOS genes (TIGR ID Os03g55800 and Os03g12500). The PCR products were gel purified, cloned into the pMD19-T vector (TaKaRa, Kusatsu, Japan), and sequenced. DNA sequences were obtained using Basic Local Alignment Search Tool (BLAST) searches (https://blast.ncbi.nlm.nih.gov/ accessed on 5 November 2020). Amino acid sequences were deduced and analyzed using DNAMAN (https://www.lynnon.com/ accessed on 5 November 2020).

### 4.3. Quantitative Real-Time PCR

Total RNA was isolated using SV Total RNA Isolation System (Promega, Madison, WI, USA) following the manufacturer’s instructions. cDNA was synthesized from 1 μg of each total RNA sample, using the Prime-Script^TM^ RT-PCR Kit (TaKaRa, Kusatsu, Japan). The qRT-PCR assay was performed on the ABI PRISM sequence detection system (Applied Biosystem, Foster City, CA, USA) using a Premix EX Taq^TM^ Kit (TaKaRa, Kusatsu, Japan). The expression level of target gene was normalized to the rice actin gene *OsACT* (TIGR ID Os03g50885). The primers and probes used for qRT-PCR analysis in this study are provided in Table S2. Five independent biological replicates were carried out.

### 4.4. Generation and Characterization of as-aos Transgenic Lines

A 728-bp (1072–1799) fragment of *OsAOS1* and a 447-bp (1396–1842) fragment of *OsAOS2* were cloned and inserted into the pCAMBIA-1301 transformation vector individually to obtain two antisense constructs. Both vectors were inserted into the Xiushui 11 plants via *Agrobacterium tumefaciens*-mediated transformation. Rice transformation, screening of the homozygous T_2_ plants, and identification of the number of insertions were performed following the same method as described previously [25]. Three T_2_ homozygous lines of as-*aos1* (as1-3, as1-5, and as1-10) and three lines of as-*aos2* (as2-10, as2-20, and as2-58), each with single insert, were used in subsequent experiments.

### 4.5. Plant Treatments

For SSB treatment, individual plant bases were infested with a third-instar larva of SSB that had been starved for 2 h before the experiment. Control plants were not manipulated (Con). For BPH treatment, individual plant bases were confined in the glass cylinders (diameter 4 cm, height 8 cm, with 48 small holes, diameter 0.8 mm; Figure S9) into which 13 gravid BPH females were released. Plants confined to empty cylinders were used as controls (Non-infested). Mechanically wounded plants were individually pricked 200 times with a needle on the low side of their leaf sheaths (W). Non-manipulated plants were used as controls (Con). For JA and SA treatments, plants were individually sprayed with 2 mL of JA (100 μg mL^−1^) or SA (70 μg mL^−1^) solution in 50 mM sodium phosphate buffer using the same method as described previously [25]. Control plants were sprayed with 2 mL of the buffer (BUF).

### 4.6. JA, SA and H_2_O_2_ Analysis

WT, as-*aos1*, and as-*aos2* plants were randomly assigned to control, SSB, and BPH treatments. For JA and SA analysis, rice leaf sheaths were harvested at 0, 1.5, and 3 h, and at 0, 3, 8, 24, and 48 h after infestation with SSB and BPH, respectively. JA and SA were extracted with ethyl acetate spiked with labeled internal standards and analyzed by the high performance liquid chromatography combined with tandem mass spectrometry (HPLC/MS/MS) system, as described previously [37]. Each treatment at each time interval was replicated five times.

For H_2_O_2_ analysis, leaf sheaths were harvested at 0, 8, and 24 h after gravid BPH female infestation. H_2_O_2_ concentrations were determined as described [38]. Each treatment at each time interval was replicated five times.

### 4.7. Analysis of TrypPI Activity

WT, as-*aos1*, and as-*aos2* plants were randomly assigned to SSB and JA+SSB treatment. For JA + SSB treatment, the plants were treated with JA for 1 day as stated above, then infested by a third-instar SSB larvae. Leaf sheaths (0.12–0.15 g) of each plant were harvested 3 days after the start of SSB infestation. TrypPI levels were measured using a radial diffusion assay as described in van Dam et al. [39]. Each treatment at each time interval was replicated five times.

### 4.8. Collection, Isolation and Identification of Rice Volatiles

Volatiles emitted were collected from individual plants (one plant per pot) of each line that was infested with SSB for 24 h or non-manipulated plants. The compounds were expressed as percentages of peak areas relative to the internal standard (IS, diethyl sebacate) per 8 h of trapping one plant. The collection, isolation, and identification of rice volatile were carried out as described in Lou et al. [40]. Collections were replicated five times for each treatment.

### 4.9. Herbivore Bioassays

Three freshly hatched SSB larvae were allowed to feed on each WT, as-*aos1*, and as-*aos2* plant or on each WT, as-*aos1*, and as-*aos2* plant that was treated for 1 day with JA, as stated above. Larval mass (to an accuracy of 0.1 mg) was measured 12 days after the start of the experiment. Thirty plants were used for each line or treatment. To explore the difference in the tolerance of WT, as-*aos1*, and as-*aos2* lines to SSB attack, plants of WT, as-*aos1*, and as-*aos2* lines were individually infested with one third-instar SSB larva. The damage levels of plants were checked, and photographs were taken daily.

To determine the colonization and oviposition behavior of BPH females, pots with two plants (a transgenic and a WT plant) were individually confined in glass cylinders. Each cylinder received 13 gravid BPH. The number of BPH on each plant was counted at 1, 2, 4, 8, 24, and 48 h after their release, and 48 h later, the insects were removed and the eggs on each plant counted under a microscope. The experiments were replicated ten times. The survival rates of BPH nymphs on WT and transgenic plants were also investigated. Plants were individually confined with the glass cylinders, into which 10 BPH neonates were introduced. The numbers of surviving insects on each plant were recorded each day until 12 days after the release of the herbivore. Ten independent replications were performed.

### 4.10. Data Analysis

The differences in expression levels of genes after various treatments, and the colonization and oviposition behavior of BPH on various lines were analyzed using Student’s *t*-test. The differences in JA, SA, H_2_O_2_, TrypPI and volatiles levels, SSB mass, and BPH survival rate were compared using one-way analysis of variance (ANOVA) followed by Tukey’s honest significant difference (HSD) post-hoc test. Data were analyzed with Statistica (Statistica, SAS Institute Inc., Cary, NC, USA).

## Data Availability

The data presented in this study are available on request from the corresponding author.

## Supplementary Materials

The following are available online at https://www.mdpi.com/2223-7747/10/3/442/s1. Table S1. Volatile compounds emitted from non-manipulated and SSB-infested plants (24 h) of as-*aos1*, as-*aos2*, and wild-type lines. Table S2. Primers and probes used in this study. Figure S1. The nucleotide and deduced amino acid sequence of *OsAOS1* and *OsAOS2*. Figure S2. Alignment of the nucleotide and amino acid sequence of OsAOS1 and OsAOS2. Figure S3. Expression levels of *OsAOS1* and *OsAOS2* in rice plants that were treated with SA. Figure S4. The transformation vector used to generate the as-*aos1* and *as-aos2* lines. Figure S5. DNA gel-blot analysis of as-*aos1* and as-*aos2* plants. Figure S6. Expression levels of *OsAOS1* and *OsAOS2* in rice plants that were infested by SSB. Figure S7. Expression levels of *OsAOS1* and *OsAOS2* in as-*aos1*, as-*aos2*, and WT plants that were infested by SSB. Figure S8. Growth phenotypes of as-*aos1*, as-*aos2*, and WT plants at one-week-old seedling stage, tillering stage, and heading stage. Figure S9. The setup used for herbivore bioassays.
