# Supplementary material for: Both Allene Oxide Synthases Genes Are Involved in the Biosynthesis of Herbivore-Induced Jasmonic Acid and Herbivore Resistance in Rice
Source: Plants (Basel). 2021 Feb 26;10(3):442. doi: 10.3390/plants10030442 (PMC7996763; doi:10.3390/plants10030442)
Supplement: Supplementary file 1 [file plants-10-00442-s001.pdf]

plants

Supplementary data

# Both Allene Oxide Synthases genes are Involved in the Biosynthesis of Herbivore-Induced Jasmonic Acid and Herbivore Resistance in rice

Jiamei Zeng[1,†], Tongfang Zhang[1,2,†], Jiayi Huangfu[1], Ran Li[1] and Yonggen Lou[1,*]

1 State Key Laboratory of Rice Biology & Ministry of Agriculture Key Lab of Molecular Biology of Crop Pathogens and Insects, Institute of Insect Sciences, Zhejiang University, Hangzhou 310058, China; tsengchiamei@zju.edu.cn (J.Z.); luckyfjy@163.com (J. H.); rli05@zju.edu.cn (R. L.)
2 College of Food Science, Southwest University, Chongqing 400715, China; zhangtf@swu.edu.cn (T. Z.)
* Correspondence: yglou@zju.edu.cn; Tel.: 0086-571-88982622
† These authors contributed equally to this work.

Table S1. Volatile compounds emitted from non-manipulated and SSB-infested plants (24 h) of as-*aos1*, as-*aos2* and wild-type plants.

Table S2. Primers and probes used in this study.

Figure S1. The nucleotide and deduced amino acid sequence of *OsAOS1* and *OsAOS2*.

Figure S2. Alignment of the nucleotide and amino acid sequence of OsAOS1 and OsAOS2.

Figure S3. Expression levels of *OsAOS1* and *OsAOS2* in rice plants that were treated with SA.

Figure S4. The transformation vector used to generate as-*aos1* and as-*aos2* lines.

Figure S5. DNA gel-blot analysis of as-*aos1* and as-*aos2* lines.

Figure S6. Expression levels of *OsAOS1* and *OsAOS2* in as-*aos1*, as-*aos2* and wild-type plants that were infested by SSB.

Figure S7. Expression levels of *OsAOS1* and *OsAOS2* in as-*aos1*, as-*aos2* and WT plants that were infested by SSB.

Figure S8. Growth phenotypes of as-*aos1*, as-*aos2* and WT plants at one-week-old seedling stage, tillering stage and heading stage.

Figure S9. The setup used for herbivore bioassays.

plants MDPI

Table S1. Volatile compounds emitted from non-manipulated and SSB-infested plants (24 h) of as-*aos1*, as-*aos2* and wild-type lines. Data represent mean amount (% of internal standard peak area, ±SE) of five replications. Letters in the same row indicate significant differences among treatments ($P < 0.05$, Tukey's HSD post-hoc test).

No.	Chemical	WT	as1-5	as2-20	WT+SSB	as1-5+SSB	as2-20+SSB
1	2-heptanone	3.52±0.82b	1.07±0.05b	2.64±1.85b	41.39±7.63a	36.59±12.05a	19.8±7.37ab
2	2-heptanol	2.68±0.14c	2.83±0.94c	1.81±0.96c	34.06±4.65a	15.75±4.01b	12.76±4.58b
3	α-thujene	0.63±0.25bc	0.40±0.19c	0.55±0.36c	3.06±0.45a	0.81±0.33bc	0.86±0.4bc
4	α-pinene	0.71±0.33a	0.18±0.1a	0.66±0.49a	0.45±0.23a	0.79±0.34a	0.58±0.24a
5	myrcene	5.53±1.42a	4.03±0.68a	3.12±1.17a	4.03±0.42a	5.23±0.58a	4.44±0.88a
6	(+)-limonene	8.74±1.1c	9.57±6.17bc	6.09±3.41c	21.39±3.83a	22.74±3.52a	10.89±0.58bc
7	(E)-linalool oxide	0.95±0.19b	1.78±1.56ab	0.86±0.12b	7.29±1.75a	4.76±1.76ab	3.63±1.25ab
8	linalool	12.93±6.8bc	4.28±0.12c	7.47±1.15bc	87.45±14.05a	61.99±23.6ab	28.72±6.28bc
9	methyl salicylate	1.68±0.79a	0.89±0.14a	1.11±0.19a	2±1.03a	2.39±0.8a	2.49±1.24a
10	unknown 1	3.08±1.08a	1.56±0.12a	5.03±2.07a	1.21±0.33a	0.95±0.24a	0.45±0.05a
11	unknown 2	0.77±0.54a	0.69±0.01a	1.35±0.49a	2.22±0.56a	4.16±2.36a	2.89±1.47a
12	α-copaene	4.62±1.65b	3.79±1.56b	2.94±1.48b	11.11±2.44a	4.66±1.19b	4.87±1.04b
13	n-tetradecane	0.35±0.08b	0.21±0.13b	0.37±0.04b	33.40a	1.45±0.49b	1.19±0.59b
14	sesquithujene	2.05±1.18a	1.5±0.35a	3.58±1.58a	3±0.8a	2.79±0.57a	11.81±6.66a
15	(-)-α-cedrene	1.66±0.56a	0.94±0.21a	2.86±1.07a	1.21±0.25a	1.3±0.23a	9.46±5.42a
16	(E)-β-caryophyllene	4.86±0.87b	1.95±0.16b	1.92±0.52b	16.2±1.16a	5.72±1.48b	5.9±1.23b
17	(E)-α-bergamotene	2.09±1.46ab	2.15±1.22ab	0.97±0.2b	9.68±3.23ab	10.34±2.46a	5.06±0.99ab
18	sesquisabinene	2.14±1.47a	1.9±1.09a	2.99±1.05a	8.66±2.77a	8.62±2.02a	5.84±1.83a
19	(E)-β-farnesene	1.98±0.39a	1.68±0.16a	3.17±1.27a	3.98±1.29a	3.57±0.94a	4.02±1.85a
20	ar-curcumene	3.53±2.47a	2.62±0.64a	3.04±1.1a	6.88±1.75a	7.41±1.27a	12.22±5.32a
21	Zingiberene	6.81±2.47a	3.77±1.32a	12.81±7.69a	22.54±7.63a	19.73±5.38a	12.1±3.42a
22	β-bisabolene	5.06±3.49a	2.86±0.37a	5.82±2.61a	13.08±4.08a	12.27±2.94a	20.4±9.6a
23	β-sesquiphellandren	5.99±3.49a	3.75±0.75a	2.79±0.24a	18.64±5.98a	18.82±4.38a	11.64±2.98a
24	(E)-γ-bisabolene	2.94±1.29a	2.52±1.87a	2.25±1.05b	10.4±3.51a	7.9±2.46a	2.54±1.99a
Total		85.3±34.33	56.92±19.91	76.2±32.16	363.33±69.82	260.74±75.4	194.56±67.26

Table S2. Primers and probes used in this study

Gene name	TIGR ID	Description	Forward primer (5'-…-3')	Reverse primer (5'-…-3')	Probe (5'-…-3')
OsAOS1	Os03g55800.1	RT-PCR	GCTAGTAGCTAGCTCGGGGA	CAGTGCAACTCCGTATCCGT	
OsAOS2	Os03g12500.1	RT-PCR	TTTGCCATCGTGGACACACT	GCTATGTACGTGGGGGAAGG	
OsAOS1	Os03g55800.1	QRT-PCR	TGATCACCAAGTGGGTGCTG	CGAGTGGAGGAGCGTGTCC	CTCAGCCCGCTGCTCAGCCTC
OsAOS2	Os03g12500.1	QRT-PCR	TGCCCATGATCATCGAGGAT	TGTAGTCGGAGCTGATGAGGAA	CTCCTCCACACGCTGCCGCTG
OsACT	Os03g50885	QRT-PCR	TGGACAGGTTATCACCATTGGT	CCGCAGCTTCCATTCCTATG	CGTTTCCGCTGCCCTGAGGTCC

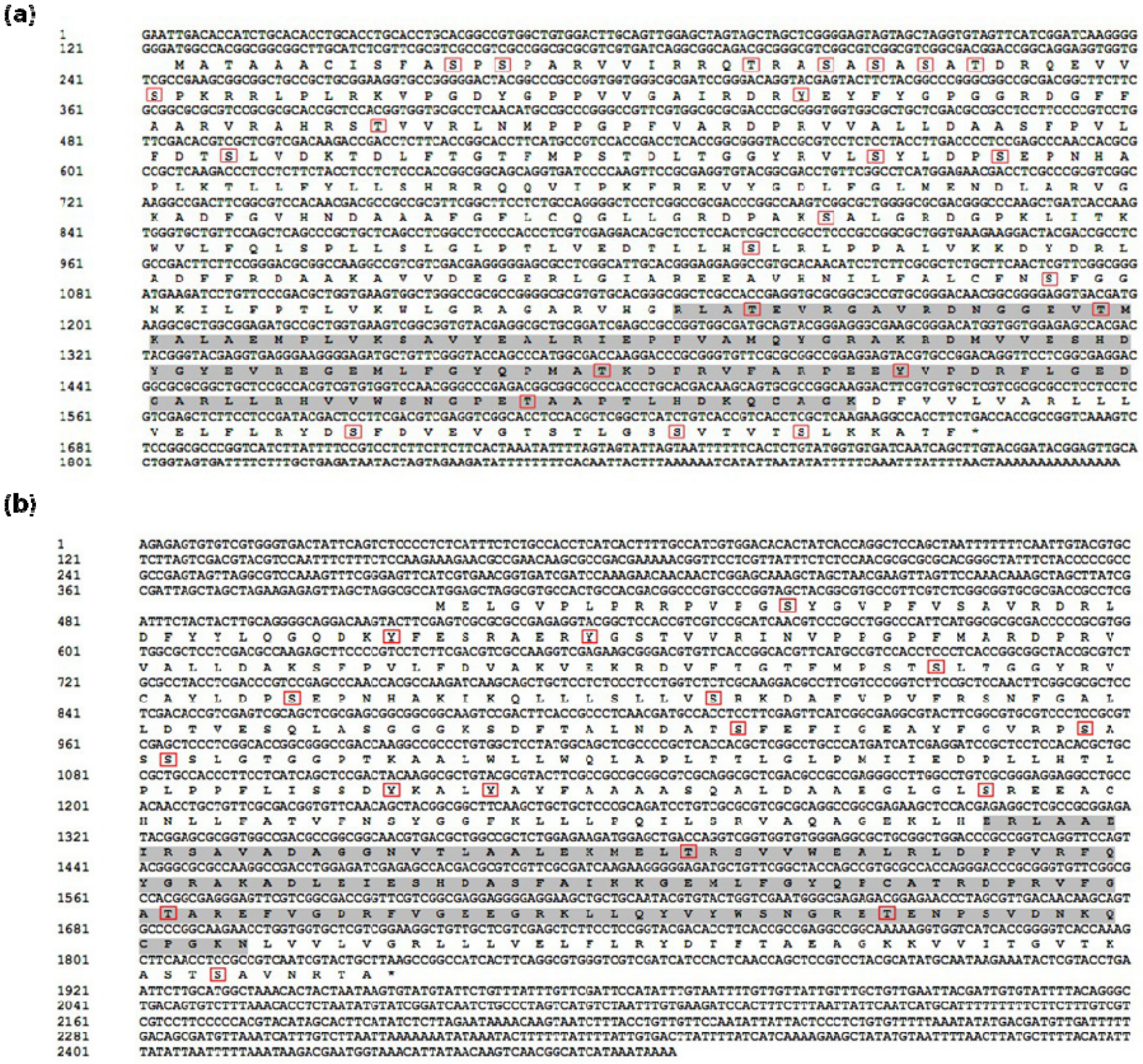

Figure S1. The nucleotide and deduced amino acid sequence of OsAOS1 (**a**) and OsAOS2 (**b**). Gray shading indicted P450 family, red frame indicted phosphorylation site.

(a)

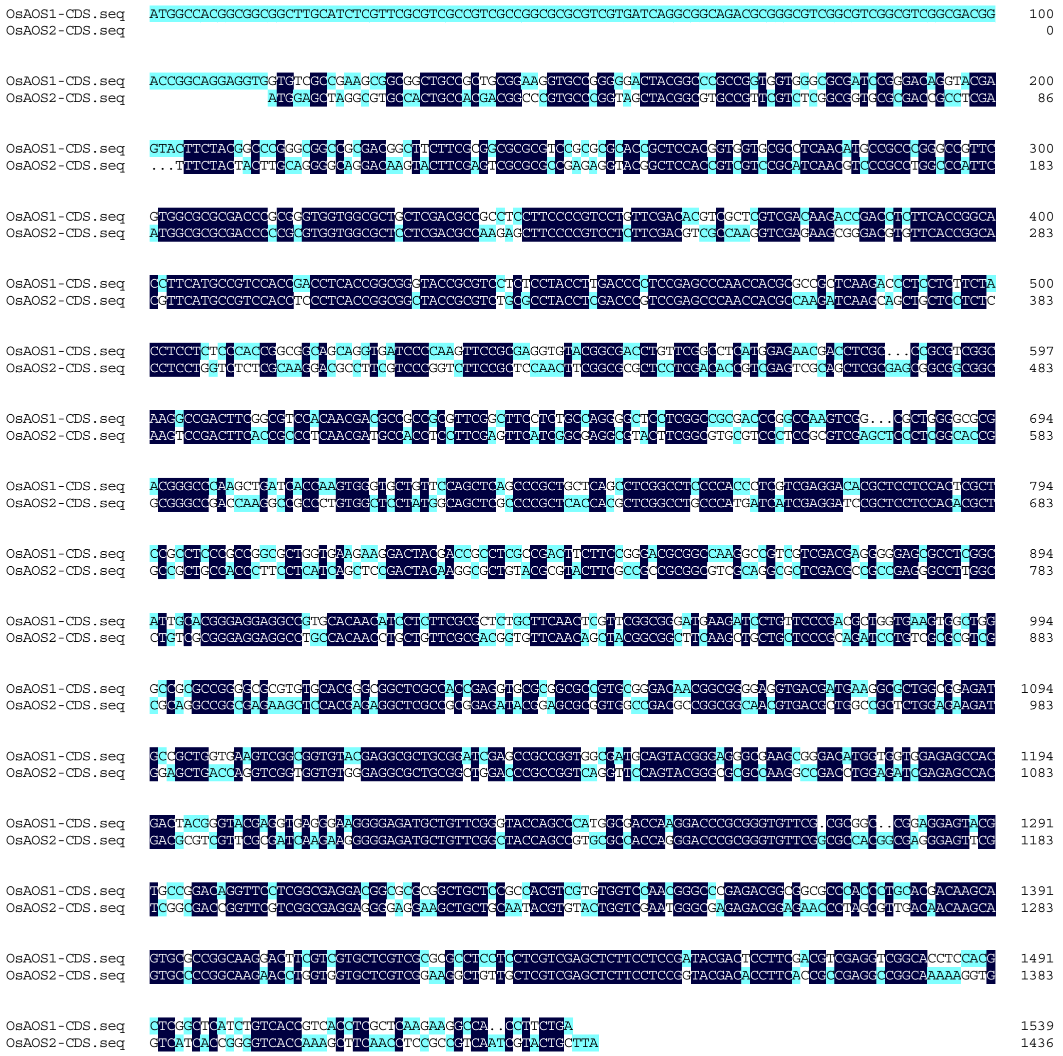

(b)

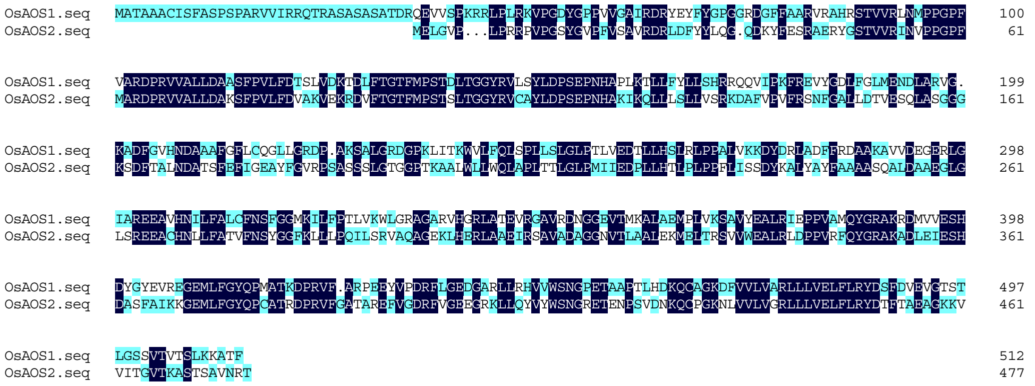

Figure S2. Alignment of the nucleotide (**a**) and amino acid (**b**) sequence of OsAOS1 and OsAOS2. The blue shading indicates the bases that are identical in the two sequences.

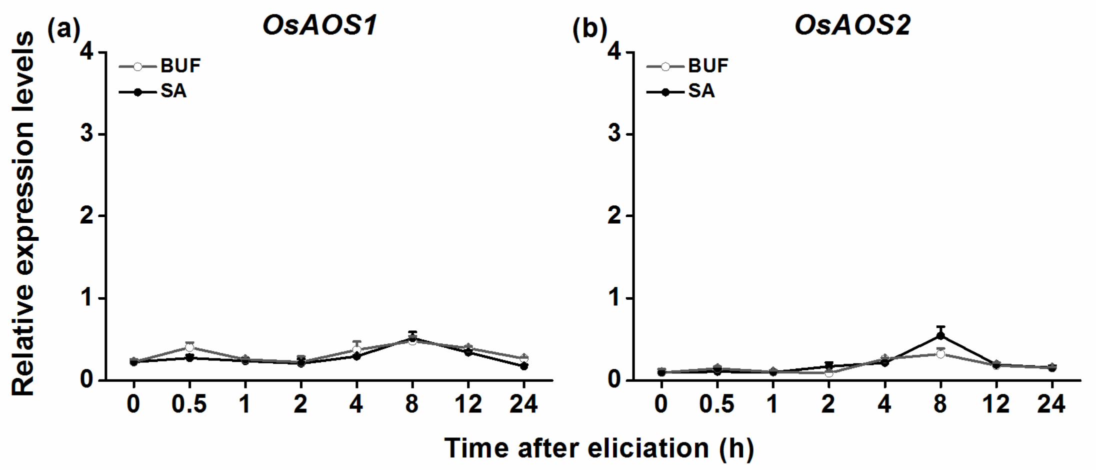

Figure S3. Expression levels of *OsAOS1* and *OsAOS2* in rice plants that were treated with SA. Mean expression levels (relative to expression levels of *OsACT*, +SE, *n* = 5) of *OsAOS1* (**a**) and *OsAOS2* (**b**) in rice leaf sheaths that were treated by buffer (BUF) or salicylic acid (SA).

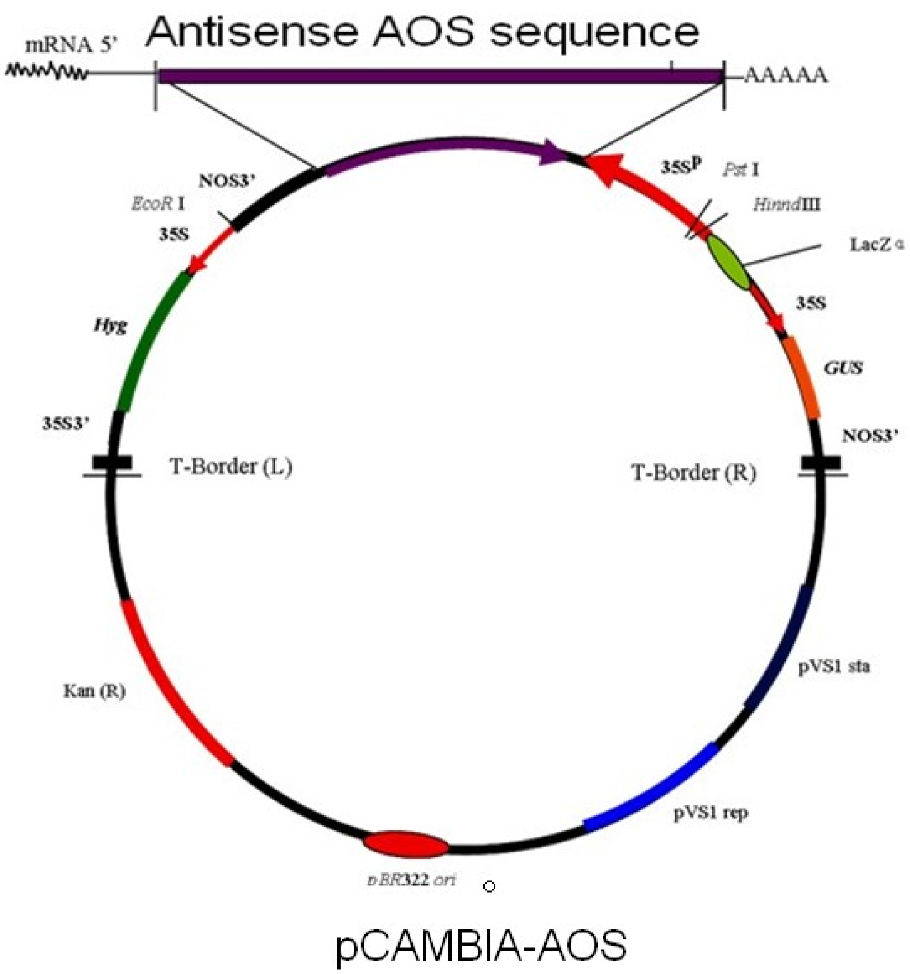

Figure S4. The transformation vector used to generate the as-*aos1* and as-*aos2* lines.

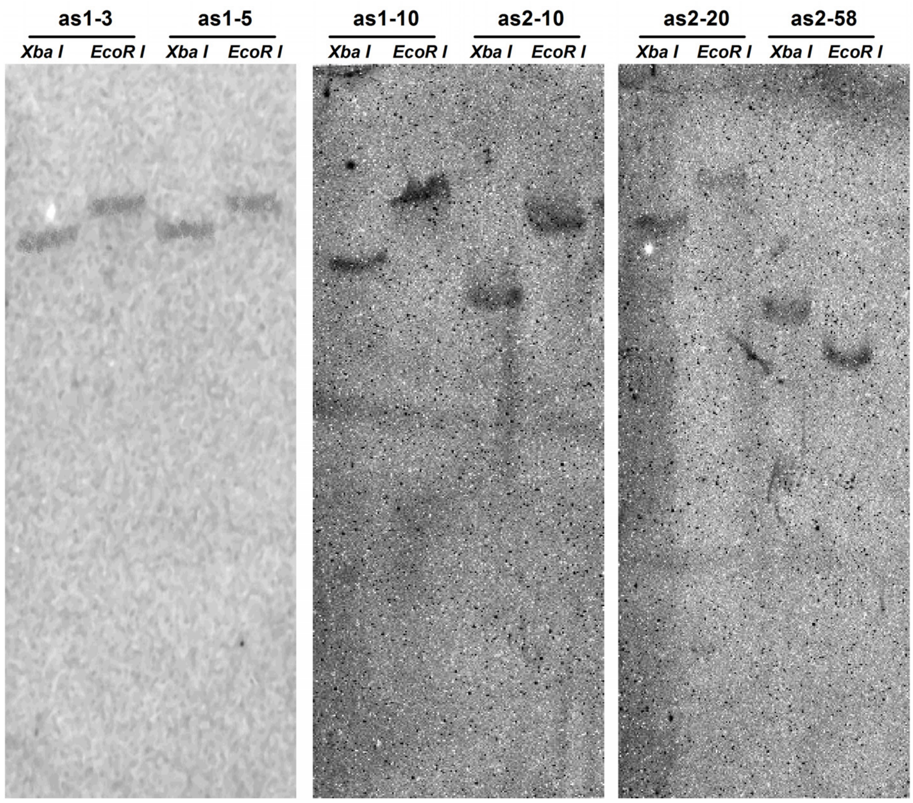

Figure S5. DNA gel-blot analysis of as-*aos1* and as-*aos2* lines plants. Genomic DNA was digested with *Xba*I or *Eco*RI. The blot was hybridized with a probe specific for reporter gene *gus*. Hybridization was created using the DIG High Prime DNA Labeling and Detection Starter Kit II (Roche). All as-*aos* lines have a single insertion of the transgene.

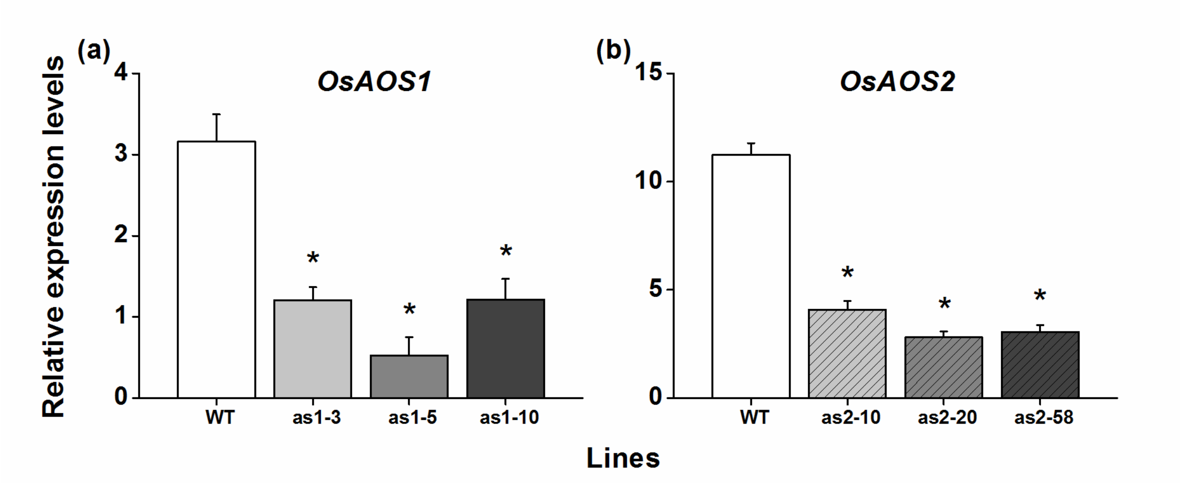

Figure S6. Expression levels of *OsAOS1* and *OsAOS2* in as-*aos1*, as-*aos2* and wild-type plants that were infested by SSB. (a) Mean transcript levels (relative to expression levels of *OsACT*, +SE, n = 5) of *OsAOS1* in as-*aos1* lines and WT plants that were individually infested by SSB for 1 h. (b) Mean transcript levels (relative to expression levels of *OsACT*, +SE, n = 5) of *OsAOS2* in as-*aos2* lines and WT plants that were individually infested by SSB for 1 h. Asterisks indicate significant differences between treatments and controls (*$P < 0.05$, Tukey's HSD post-hoc test).

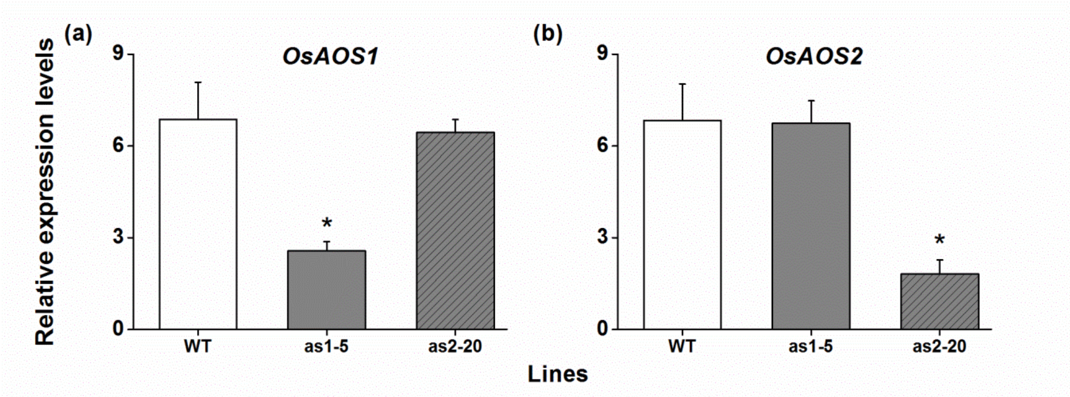

Figure S7. Expression levels of *OsAOS1* and *OsAOS2* in as-*aos1*, as-*aos2* and WT plants that were infested by SSB. Mean transcript levels (relative to expression levels of *OsACT*, +SE, *n* = 5) of *OsAOS1* (**a**) or *OsAOS2* (**b**) in as-*aos1* (as1-5), as-*aos2* (as2-20) and WT plants that were individually infested by SSB for 3 h. Asterisks indicate significant differences in as-*aos* lines compared with WT plants (**P* < 0.05, Tukey's HSD post-hoc test).

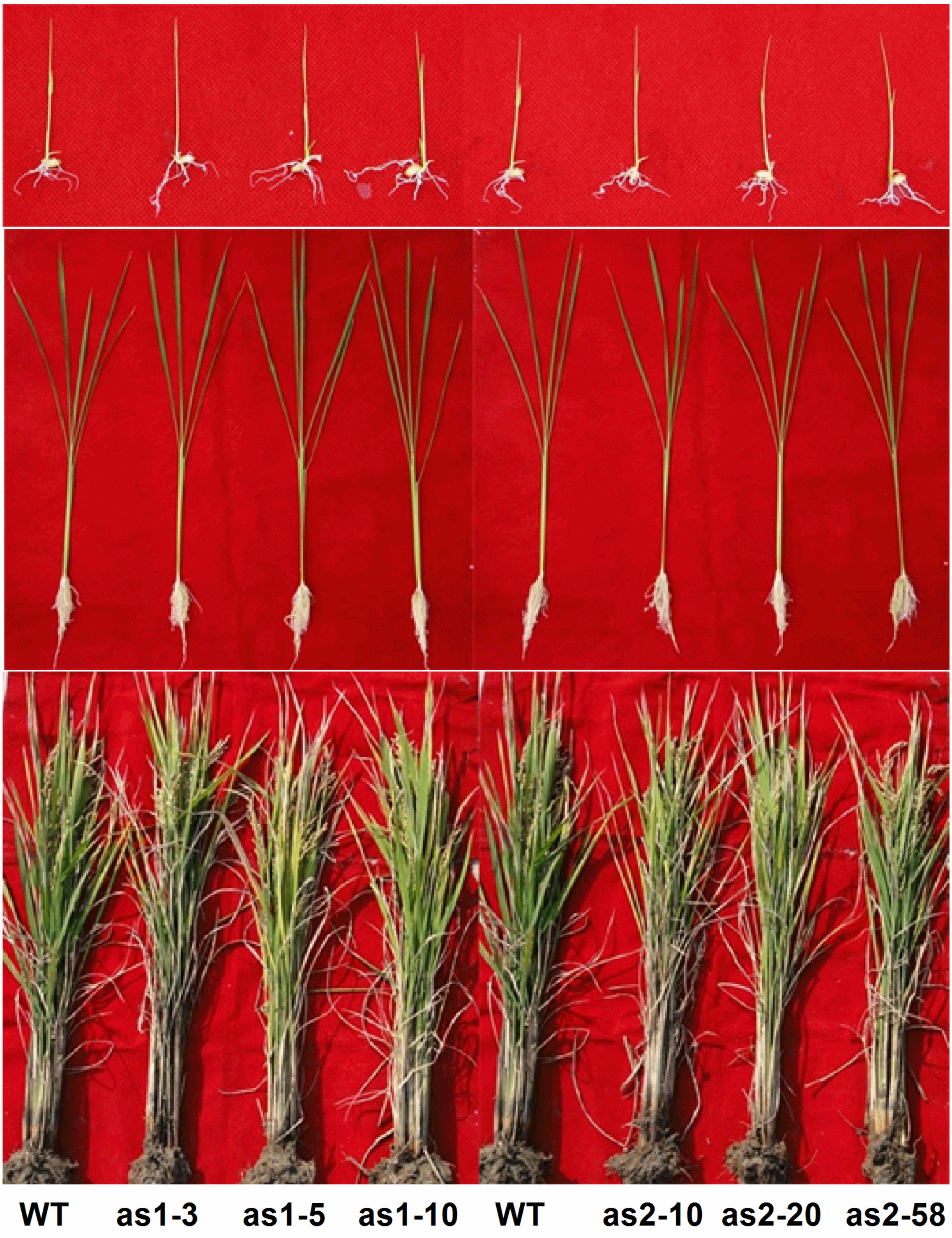

Figure S8. Growth phenotypes of as-*aos1*, as-*aos2* and WT plants at one-week-old seedling stage, tillering stage and heading stage.

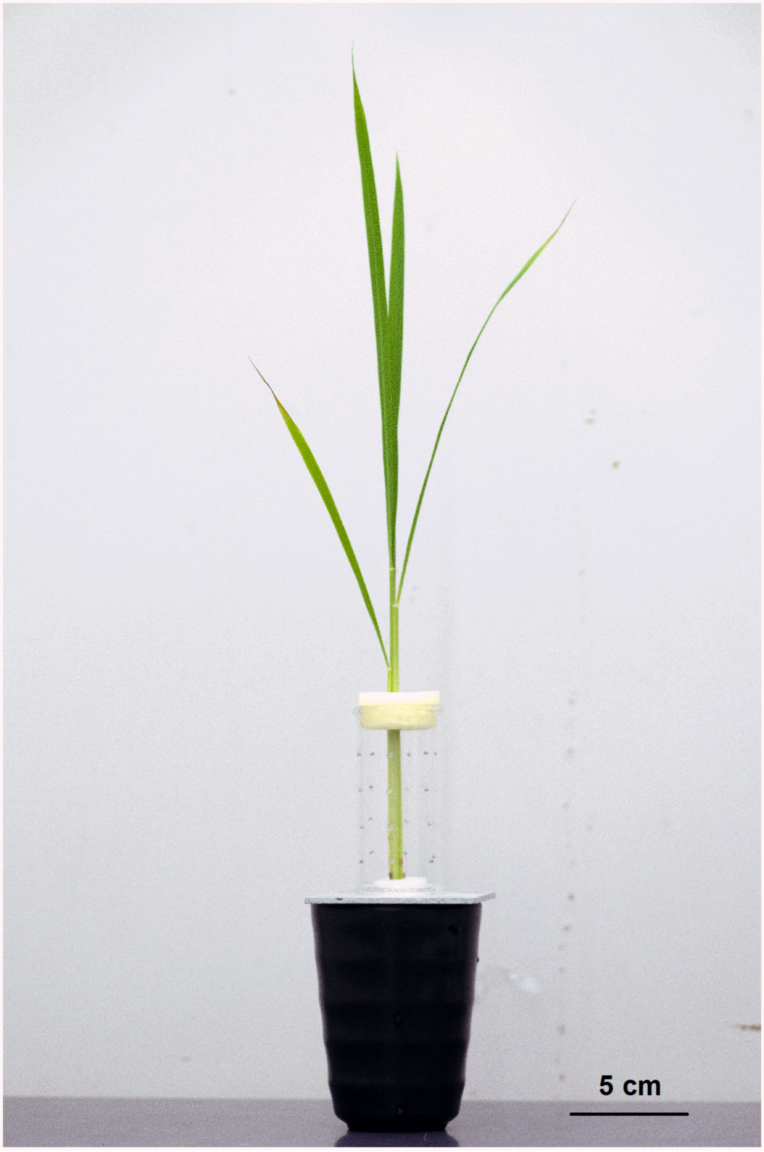

Figure S9. The setup used for herbivore bioassays.
